# Real-time imaging of cGMP signals in platelets

**DOI:** 10.1186/2050-6511-16-S1-A98

**Published:** 2015-09-02

**Authors:** Lai Wen, Martin Thunemann, Kjestine Schmidt, Cor de Wit, Susanne Feil, Marcus Olbrich, Harald Langer, Meinrad Gawaz, Robert Feil

**Affiliations:** 1 Interfakultäres Institut für Biochemie, University of Tübingen, Tübingen, Germany; 2 Institut für Physiologie, Universität zu Lübeck, Lübeck, Germany; 3 Department of Cardiology & Cardiovascular Medicine, University of Tübingen, Tübingen, Germany

## Background

cGMP plays an essential role in platelet aggregation. However, the intracellular cGMP concentrations and the spatiotemporal dynamics of cGMP signals in platelets during hemostasis and thrombosis are largely unknown. To visualize cGMP signals *in vivo*, we have generated so-called cGMP sensor knock-in mice [1]. A Cre recombinase-activatable expression cassette encoding the fluorescence resonance energy transfer (FRET)-based cGMP sensor, cGi500, driven by the CAG promoter was integrated into the Rosa26 locus. Depending on the strategy to activate sensor expression, these mice can show either ubiquitous or tissue-specific sensor expression allowing for delineation of cGMP signaling in live cells *in vitro* and *in vivo*.

## Results

cGMP signaling was dissected through real-time FRET imaging of activated mouse platelet thrombi *in vitro* in a flow chamber system. We found that NO triggers fast cGMP signals in platelet thrombi under flow, and that PDE2, 3 and 5 are responsible for cGMP degradation. Simultaneous imaging of cGMP and Ca^2+^ revealed that the concentration of these two second messengers has an inverse relationship. NO-induced cGMP signals suppress procoagulant Ca^2+^ signals, indicating an inhibitory effect of cGMP on platelet aggregation under these conditions. Moreover, using a mouse model of mechanical injury-induced thrombosis in the arterioles of the cremaster muscle, intravital FRET imaging showed that endogenous cGMP signals in platelets increase robustly during thrombus growth *in vivo*.

## Conclusion

Taken together, our data demonstrate that the cGMP sensor knock-in mice are useful tools for real-time monitoring of cGMP signals in platelets. cGMP may act as a brake to prevent the platelet thrombus from overgrowth and vessel occlusion via cGMP/Ca^2+^ signaling.
